# Circular RNA expression profile and its potential regulative role in human abdominal aortic aneurysm

**DOI:** 10.1186/s12872-020-01374-8

**Published:** 2020-02-10

**Authors:** Min Zhou, Zhenyu Shi, Liang Cai, Xu Li, Yong Ding, Tianchen Xie, Weiguo Fu

**Affiliations:** Department of Vascular Surgery, Zhongshan Hospital, Fudan University, Shanghai, 180 Fenglin Road, Shanghai, 200032 China

**Keywords:** Circular RNA, Abdominal aortic aneurysm, Microarray, Pathogenesis, Therapeutic targets

## Abstract

**Background:**

This study aimed to identify the differentially expressed circular RNAs (circRNAs) between human abdominal aortic aneurysm (AAA) and the control group.

**Methods:**

High-throughput sequencing was applied to determine the circRNA expression profiles of 4 paired aortic samples. Real-time quantitative reverse transcription-polymerase chain reaction (qRT-PCR) was carried out to testify 6 randomly selected dysregulated circRNAs. Kyoto Encyclopedia of Genes and Genomes and Gene ontology (GO) analysis were conducted for functional annotation of the parental genes. Additionally, interaction networks between circRNA and 5 putative microRNA (miRNA) partners were constructed.

**Results:**

Finally, 411 differentially expressed circRNAs were discovered, including 266 downregulated and 145 upregulated circRNAs. Compared with the control group, the expression level of hsa (*Homo sapiens*) _circ_0005360 (LDLR) and hsa_circ_0002168 (TMEM189) were proved significantly lower in the AAA group by qRT-PCR. Regarding upregulated circRNAs, the most enriched GO molecular function, biological process and cellular component terms were poly(A) RNA binding, negative regulation of transcription from RNA polymerase II promoter and nucleoplasm, respectively. Moreover, circRNA/miRNA interaction networks showed that hsa_circ_0005360/miR-181b and hsa_circ_0002168/miR-15a axis might have a regulative role in human AAA.

**Conclusions:**

This study revealed new circRNAs potentially related to the pathogenesis of AAA. Further experimental studies are warranted to clarify the potential molecular mechanisms.

## Background

Ruptured abdominal aortic aneurysm (AAA) is an important cause of cardiovascular mortality in men over the age of 65 years [1]. In Sweden, developed screening program had only a minor effect on AAA mortality [2]. Currently, little is known about the pathobiology and underlying molecular mechanism of AAA, which limits the development of medical treatments to stabilize aneurysms. However, genetic component provides with a new orientation in the etiology of AAA. Determination of the aberrant genes related with AAA is an established approach to expanding the knowledge of the pathways contributing to aneurysmal degeneration of the abdominal aorta [3].

Circular RNAs (circRNAs) represent a new type of endogenous non-coding RNAs produced by non-colinear reverse splicing. They are generated by an incorporation of the 3′ end and 5′ end and highly stable in vivo because of covalently closed loop structures [4]. Numerous studies have determined that circRNAs can regulate transcriptional and post-transcriptional gene expression [5, 6]. The classic pathway is circRNA-miRNA (microRNA)-mRNA (messenger RNA), which indicates that circRNA binds and inhibits miRNA, subsequently affecting the target mRNA expression [7, 8]. Recently, circRNAs have been confirmed ideal candidates for diagnostic biomarkers and therapeutic targets in cardiovascular diseases [9–11]. Nevertheless, the circRNA expression profile and its potential regulative role in human AAA remain unclear.

In this study, we intended to identify the differentially expressed circRNAs between the AAA and the control group. Computational analysis was performed to predict the circRNA/miRNA interaction networks. Several dysregulated circRNAs expression levels were further testified by real-time quantitative reverse transcription-polymerase chain reaction (qRT-PCR).

## Methods

### Patients

This study was approved by the Institutional Ethics Committees of Zhongshan Hospital, Fudan University (B2018-040R) and complied with the Declaration of Helsinki. From March 2018 to September 2018, 4 consecutive patients with AAA, who were unsuitable for endovascular repair, underwent open surgery at our center. All the 4 patients received computed tomography angiography examinations preoperatively and were not found with underlying connective tissue diseases. Written informed consent was obtained from all enrolled patients and donors next-of-kin. Full thickness AAA specimens were obtained from the aneurysmal segment of abdominal aorta and stored at − 80 °C until assayed. Over the same period, abdominal aortic samples just below the aortic trunk from 4 heart-beating brain-dead organ donors were used as the controls. Clinical characteristics and maximal infrarenal aortic diameter were recorded for patients with AAA, but this information was unavailable for donors (Table 1). The median age of AAA patients and donors was 58.5 and 39 years, respectively (*P* = 0.057).

**Table 1:** Clinical characteristics of patients with abdominal aortic aneurysm and organ donors

	Patient 1	Patient 2	Patient 3	Patient 4	Control 1	Control 2	Control 3	Control 4
Sex	Male	Male	Male	Male	Male	Male	Male	Female
Hypertension	–	+	–	+	U	U	U	U
Diabetes	–	+	–	–	U	U	U	U
Dyslipidemia	–	–	–	–	U	U	U	U
CAD	–	–	–	–	U	U	U	U
COPD	–	–	–	–	U	U	U	U
Renal dysfunction	–	–	–	–	U	U	U	U
Stroke	–	–	–	+	U	U	U	U
Smoking	+	–	+	+	U	U	U	U
Maximum abdominal aortic diameter (mm)	61.7	65.1	59.7	56.0	U	U	U	U
Aortic neck diameter (mm)	33.9	20.2	21.6	20.0	U	U	U	U
Proximal landing zone (mm)	27.9	5.0	45.1	20.0	U	U	U	U
Aortic neck angulation (°)	90	30	10	15	U	U	U	U

Abbreviations: *CAD* coronary artery disease, *COPD* chronic obstructive pulmonary disease, *U* unclear

### Microarray analysis

Total RNA of the 4 paired aortic samples were isolated using TRIzol Reagent (Invitrogen, Carlsbad, CA, USA). NanoDrop ND-1000 (NanoDrop Technologies, Wilmington, DE, USA) was utilized to assess the quality and quantification of the total RNA. The OD260/280 ratios of our samples were located between 1.8 and 2.1, which were acceptable. Then, RNA integrity and genomic DNA contamination were evaluated by electrophoresis on a denaturing agarose gel. RNase R (Epicentre Inc., Madison, WI, USA) was used to degrade the linear and ribosomal RNAs.

The enriched circRNAs were amplified and transcribed into fluorescence labeling complementary RNA (cRNA) (Arraystar, Rockville, MD, USA). RNeasy Mini Kit (Qiagen, Hilden, Germany) was applied to purify the labeled cRNAs. Subsequently, the fragmentation mixture, consisting of 1 μg of each labeled cRNA, 5 μl 10× blocking agent and 1 μl 25× fragmentation buffer, was incubated at 60 °C for 30 min. The fragmented labeled cRNAs were hybridized onto the circRNA expression microarray slide (Arraystar Human circRNA Array V2). The slides were incubated for 17 h at 65 °C and then washed, fixed and scanned.

The scanned images were collected and imported into Agilent Feature Extraction software (Agilent Technologies Inc., CA, USA). Quantile normalization and subsequent data processing were carried out using the R software packages (R version 3.6.1, https://www.r-project.org/). Dysregulated circRNAs between 4 paired aortic samples were determined using the Limma package. The statistical significance was set as |log_2_ fold change (FC)| ≥ 1 with *P* value < 0.05 estimated by t-test. A scatter plot and heat map were generated to assess the variation in circRNAs expression profiles between the AAA and the control group. The dysregulated circRNAs were showed via a Volcano Plot.

### Computational bioinformatics analysis

The interaction between aberrant circRNA and potential target microRNA was predicted by miRanda and TargetScan [12, 13]. Based on the prediction of miRNA binding sites, a circRNA/miRNA interaction network between circRNAs and 5 putative miRNA partners were constructed.

For the functional annotation of parental genes of the dysregulated circRNAs, the Database for Annotation, Visualization and Integrated Discovery (DAVID; http://david.abcc.ncifcrf.gov/) was utilized to conduct Gene Ontology (GO) analysis, including molecular function (MF), biological process (BP) and cellular component (CC). Further, pathway enrichment analysis was performed by Kyoto Encyclopedia of Genes and Genomes (KEGG) (http://www.genome.jp/kegg/kegg2.html) to detect the biological pathways of the involved parental genes [14]. The significantly enriched GO terms (*P* value < 0.05) were ranked by -log_10_ (*P* value).

### qRT-PCR analysis

The total RNA of 4 paired aortic samples was extracted using the Trizol reagent (Takara Bio Inc., Kusatsu, Japan). A Reverse Transcription kit (Takara Bio Inc.) was utilized to synthesize the complementary DNA. Circular RNAs of interest were amplified according to the manufacturer’s protocol of SYBR-Green PCR Mix (Takara Bio Inc.). The PCR primer sequences are shown in Table 2. β-actin was used as the internal control. The relative expression level of circRNAs was calculated using the 2^−ΔΔCT^ equation [15].

**Table 2:** Primers used for real-time quantitative reverse transcription-polymerase chain reaction

Genes	Forward and reverse sequence	Product length (bp)
β-actin	F:5′ GTGGCCGAGGACTTTGATTG3’ R: 5′ CCTGTAACAACGCATCTCATATT3’	73
hsa_circ_0060063	F:5′ TCTAAGGTGTCAGATGCCTGATAC 3′ R:5′ TTCTCCACACAGCTAGTATACATGC 3’	110
hsa_circ_0070382	F:5’ TTCCCTACAAAGGACTCTCAGCAT 3′ R:5′ ACTTCATTGGAGTAGGTCTGTTTGG 3’	69
hsa_circ_0005360	F:5’ AGCCAGCTCTGCGTGAACCT 3′ R:5′ CGTTGTTGTCCAAGCATTCG 3’	121
hsa_circ_0002168	F:5’ GACCTACTTCTGCATCACCACAGTT 3′ R:5′ TGTCAGCACCCCAGTGTACCA 3’	80
hsa_circ_0028198	F:5’ TCAAGACAAAGAACTCCCAAATGA 3′ R:5′ AAGAGAGAATCTGCATGATACACCA 3’	81
hsa_circ_0027446	F:5’ CTGGAGAAAAACGGCCAAG 3′ R:5′ TGCTGCCTTTGGGTCTTC 3’	93

Abbreviations: hsa, *Homo sapiens*; bp, base pair; F, forward; R, reverse

### Statistical analysis

The relative expression level was compared by Student’s t-test. Two tailed *P* value < 0.05 was considered statistically significant. Statistical analyzes were carried out using Stata version 14.0 (StataCorp, College Station, Tex, USA).

## Results

### circRNA expression profiles

**Fig. 1:**
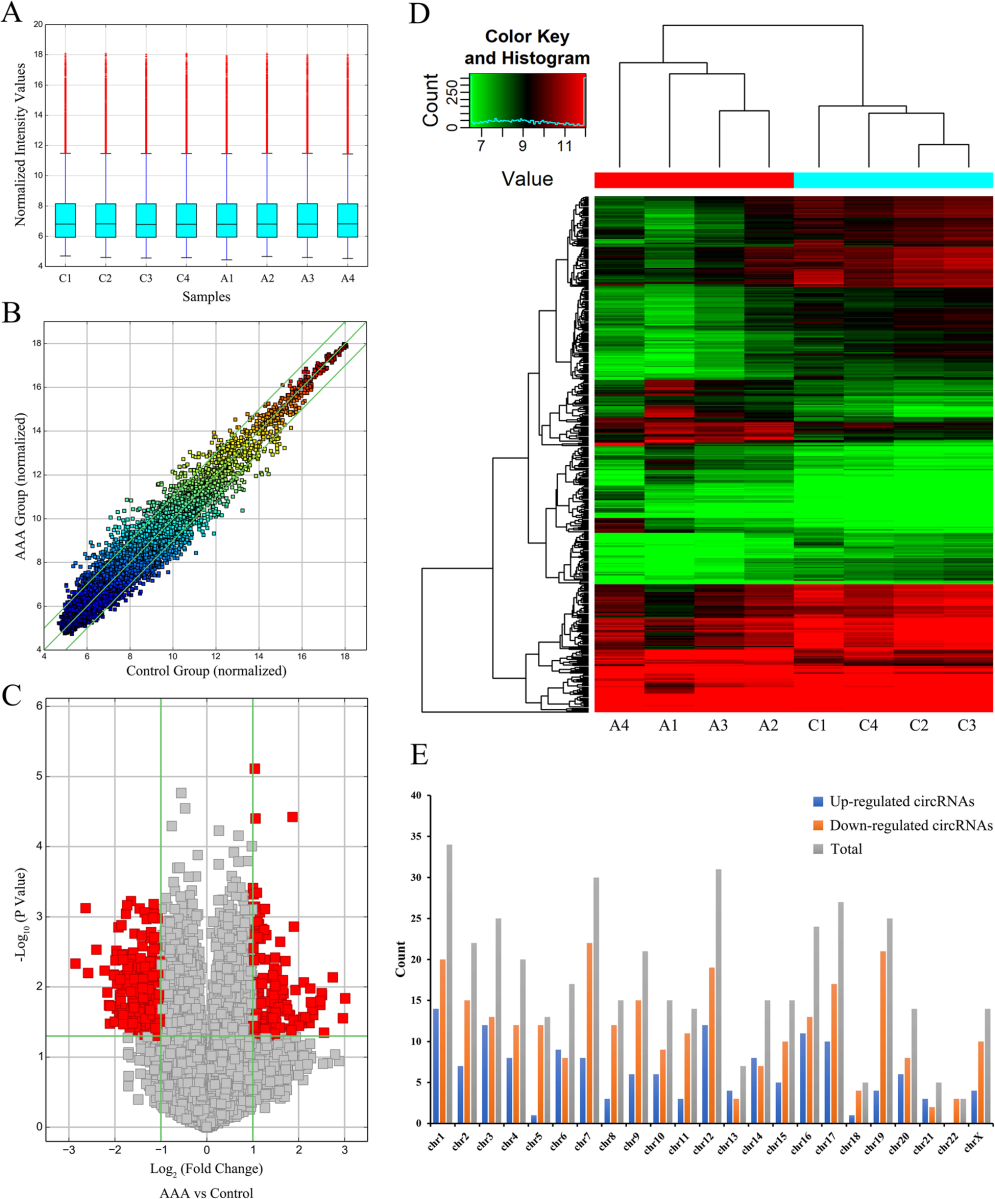
circRNAs expression profiles detected by microarray in the AAA group and control group. **a** The box plot shows the nearly identical distributions of normalized intensity values from the aortic samples of the AAA and control group. **b** The scatter plot is built to assess the expression variation of circRNAs between the two groups. The X and Y axes indicate the normalized intensity values of each circRNAs from the AAA and control group. The dots above the upper green line and below the lower green line represent the dysregulated circRNAs with a fold change (FC) > 2.0 between the two groups. **c** The volcano plot presents differentially expressed circRNAs in AAA. The vertical lines correspond to 2-fold upregulation and downregulation, the horizontal line indicates *P* value of 0.05. The red dots represent the differentially expressed circRNAs (FC > 2.0 and *P* value < 0.05). **d** Hierarchical clustering analysis reveals a distinguishable expression profile of circRNAs between the AAA and control group. Each column indicates an aortic sample, each row represents a circRNA. The red and green color indicate high and low expression level, respectively. **e** Chromosomal distribution of the differentially expressed circRNAs between the two groups

To investigate the circRNA expression profiles in human AAA, we performed the high-throughput sequencing to identify the dysregulated circRNAs. In total, 13,295 circRNAs were detected. The box plot showed the nearly identical distributions of normalized intensity values from 4 paired aortic samples (Fig. 1a). A scatter plot visualized the variation of circRNA expression profile between the two groups (Fig. 1b). The volcano plot revealed significantly aberrant circRNAs with |log_2_ FC| ≥ 1 and *P* value < 0.05 (Fig. 1c). Hierarchical clustering identified a distinct circRNA expression pattern among the samples (Fig. 1d). Finally, 411 circRNAs were observed differentially expressed, including 145 upregulated and 266 downregulated circRNAs in AAA. Based on the FC, the top 20 dysregulated circRNAs are summarized in Table 3. Among these dysregulated circRNAs, 357(86.9%) circRNAs were found exonic. The circRNA distributions among the human chromosomes were also illustrated (Fig. 1e).

**Table 3:** The top 20 dysregulated circRNAs in AAA group compared with control group

circRNA	Gene Symbol	Regulation	*P*-value	Fold Change (abs)	chrom
hsa_circ_0001588	HIST1H4E	up	0.014	8.04	chr6
hsa_circ_0000517	RPPH1	up	0.028	7.77	chr14
hsa_circ_0006156	FNDC3B	up	0.007	6.66	chr3
hsa_circ_0000518	RPPH1	up	0.044	5.85	chr14
hsa_circ_0000524	RBM23	up	0.013	5.66	chr14
hsa_circ_0007148	FNDC3B	up	0.012	5.65	chr3
hsa_circ_0068655	UBXN7	up	0.018	5.54	chr3
hsa_circ_0008285	CDYL	up	0.016	5.52	chr6
hsa_circ_0042268	ATPAF2	up	0.021	4.89	chr17
hsa_circ_0009361	GNB1	up	0.032	4.71	chr1
hsa_circ_0092291	EIF2S2	down	0.005	7.25	chr20
hsa_circ_0005073	ADPGK	down	0.001	6.22	chr15
hsa_circ_0090069	PHEX	down	0.006	5.99	chrX
hsa_circ_0057691	SATB2	down	0.003	5.30	chr2
hsa_circ_0003249	LRP11	down	0.006	4.67	chr6
hsa_circ_0092290	SCRIB	down	0.012	4.49	chr8
hsa_circ_0092371	PLEKHG4B	down	0.018	4.38	chr5
hsa_circ_0027446	HMGA2	down	0.033	4.32	chr12
hsa_circ_0074306	DIAPH1	down	0.018	4.30	chr5
hsa_circ_0008554	KANK2	down	0.007	4.07	chr19

Abbreviations: *hsa Homo sapiens*, *chr* chromosome

### Validation of dysregulated circRNAs

**Fig. 2:**
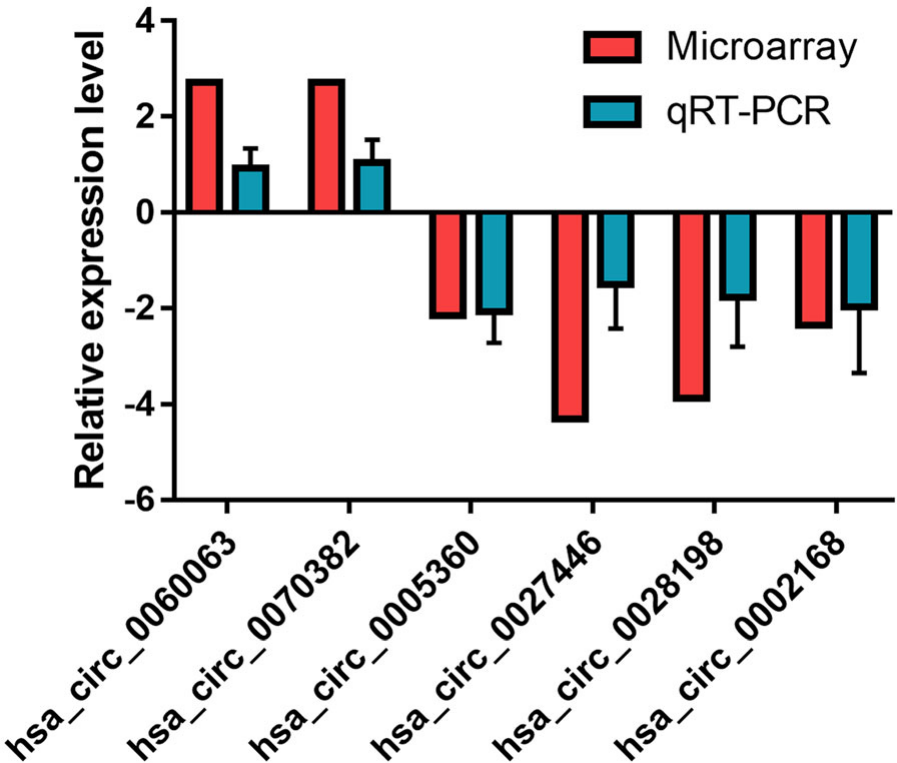
Validation of six randomly selected dysregulated circRNAs by qRT-PCR. Each circRNA was evaluated at least three times and compared with the results of microarray. The Y axis indicates the fold change of AAA vs control of each circRNA

To testify the results of microarray analysis, 6 dysregulated circRNAs (2 upregulated and 4 downregulated circRNAs), including hsa (*Homo sapiens*) _circ_0060063 (UQCC1), hsa_circ_0070382 (AFF1), hsa_circ_0027446 (HMGA2), hsa_circ_0028198 (ANAPC7), hsa_circ_0005360 (LDLR) and hsa_circ_0002168 (TMEM189), were selected for further qRT-PCR validation. Compared with the control group, the expression level of hsa_circ_0005360 (LDLR) and hsa_circ_0002168 (TMEM189) were proved significantly lower in the AAA group (Fig. 2).

### Functional analysis of parental genes

**Fig. 3:**
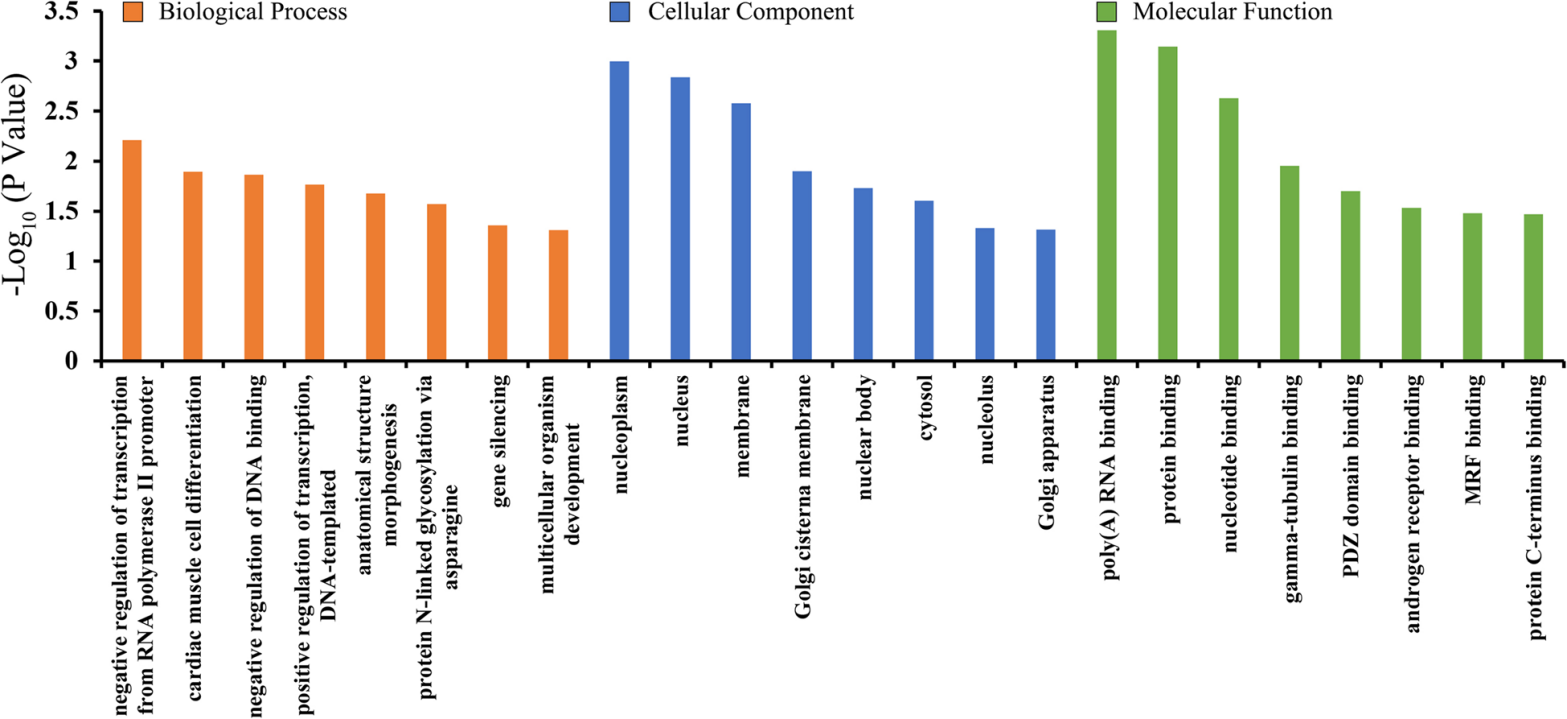
Gene ontology (GO) enrichment analysis for the parental genes of upregulated circRNAs. The X axis shows the top 8 GO terms in biological process, cellular component and molecular function corresponding to the upregulated circRNAs. The Y axis indicates log_10_ (*P* value)

For the upregulated circRNAs, the top 8 enriched GO terms were showed in Fig. 3 and ranked by -log_10_ (*P* value). As a result, the most enriched MF, BP and CC terms were poly(A) RNA binding, negative regulation of transcription from RNA polymerase II promoter and nucleoplasm, respectively. Moreover, KEGG analysis presented that only one pathway significantly related to these upregulated circRNAs (*P* = 0.020), namely transcriptional misregulation in cancer. However, the downregulated circRNAs failed to be enriched in any GO terms or KEGG pathways.

### miRNA prediction and competing endogenous RNA network construction

**Fig. 4:**
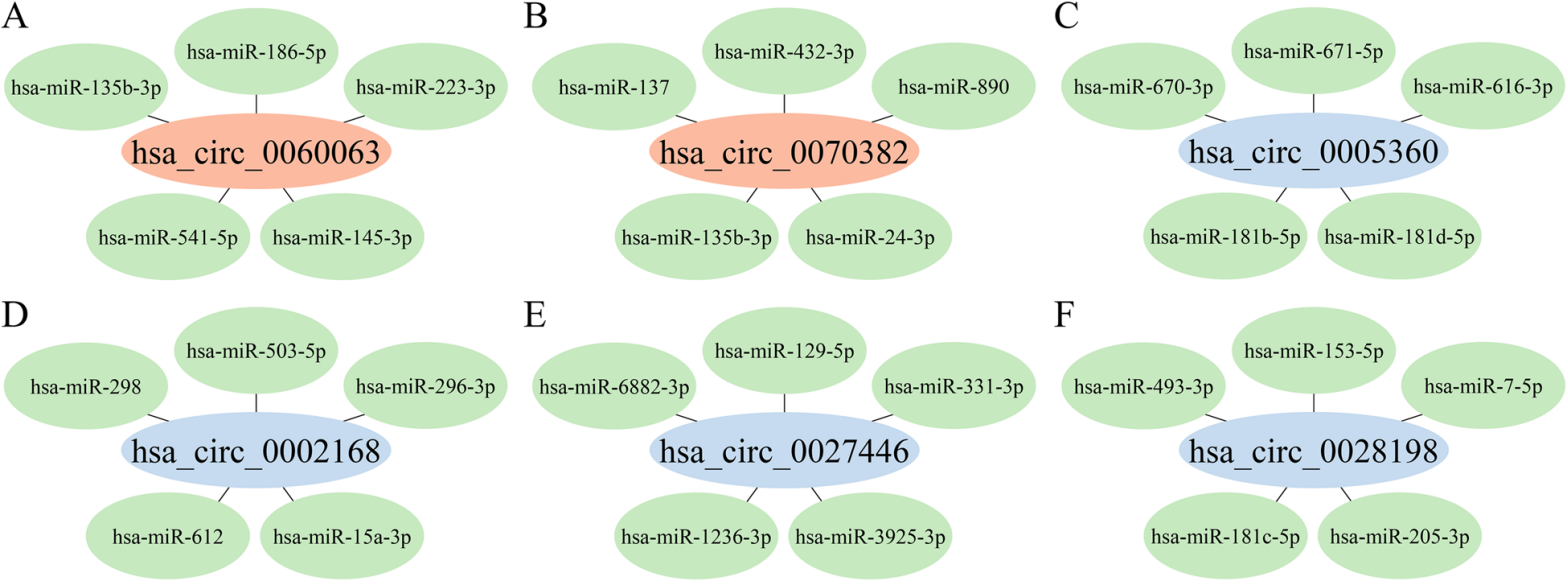
The predicted circRNA/miRNA interaction networks for six randomly selected circRNAs. **a**, **b** The red nodes indicate upregulated circRNAs. **c**-**f** The blue nodes represent downregulated circRNAs. The green nodes are five complementary binding miRNAs of each circRNA

circRNAs usually function as an inhibitor of their interacting miRNA partners. To assess the potential function of these dysregulated circRNAs, 5 putative miRNA partners were predicted for each circRNA. In total, 2055 circRNA/miRNA pairs having one or more binding regions were generated. Additionally, interaction networks between circRNA and their top 5 predicted miRNAs were constructed for the above qRT-PCR confirmed circRNAs (Fig. 4).

## Discussion

It is well established that AAAs are associated with smooth muscle cell apoptosis, local inflammatory cells infiltration, and extracellular matrix degradation in the aortic media layer at the aneurysm site [3, 16–18]. Numerous clinical studies have confirmed that several traditional cardiovascular risk factors, such as gender, age, smoking, dyslipidemia and hypertension, lead to the development of AAAs [19–21]. However, the underlying molecular mechanisms responsible for the initiation and progression of AAAs remain unclear. Using gene expression profile, several researchers detected that immune and inflammatory response had a great impact on the pathogenesis of AAA [22–24]. Recently, advances in novel computational approaches and high-throughput sequencing techniques have sparked new interest in the research on noncoding RNAs [25, 26]. As an important member of noncoding RNAs, circRNAs are abundant, stable and highly conserved. Moreover, circRNAs can function as miRNA sponges and subsequently regulate gene expression, which increase our understanding of cardiovascular diseases research [9, 10]. Thus, we utilized high-throughput sequencing to analyze the circRNA expression profile between the AAA and control group.

In this study, we identified 411 differentially expressed circRNAs, of which 145 circRNAs were significantly upregulated and 266 circRNAs were significantly downregulated in AAA samples compared with controls. Six randomly selected circRNAs, including hsa_circ_0060063, hsa_circ_0070382, hsa_circ_0027446, hsa_circ_0028198, hsa_circ_0005360 and hsa_circ_0002168, were testified by qRT-PCR. The expression level of hsa_circ_0005360 and hsa_circ_0002168 were confirmed in accordance with the microarray analysis. Specially, the parental gene of hsa_circ_0005360 is LDLR, whose variant is proved associated with AAA in a genome-wide association study based on population [27]. Moreover, LDLR-deficient mice infused with angiotensin II are widely used as animal models for AAA [28–30]. Considering that hsa_circ_0005360 is alternatively transcribed from exons of LDLR, hsa_circ_0005360 may have a potential role in AAA pathogenesis.

To further detect the regulative role of circRNAs in AAA, KEGG and GO analysis were performed for the functional annotation of parental genes. The most enriched MF, BP and CC terms were associated with poly(A) RNA binding, negative regulation of transcription from RNA polymerase II promoter and nucleoplasm, respectively. In addition, KEGG pathway analysis determined that transcriptional misregulation in cancer was the only significantly enriched pathway. These processes indicated that the parental genes of dysregulated circRNAs may participate in the transcriptional regulation of AAA. Similar with their parental gene function, circRNAs can also regulate transcriptional and posttranscriptional gene expression, especially functioning as miRNA sponges.

Previously, Zheng et al. found that hsa_circ_000595 was upregulated in human AAA tissues, which would reduce the expression of miR-19a and subsequently promote human vascular smooth muscle cells (VSMC) apoptosis [31]. Similarly, apoptosis-related circRNAs were observed altered in AAA animal models [26]. Besides, circWDR77/miR-124/FGF-2 and circSATB2/miR-939/STIM1 regulatory axis are verified to regulate VSMC proliferation and migration [32, 33]. In terms of other cell types in aorta, hsa_circ_0010729 was presented to regulate the apoptosis and proliferation of vascular endothelial cells by targeting the miR-186/HIF-1α axis [34]. These findings suggest that circRNAs can bind to miRNAs and regulate gene expression at the posttranscriptional level, which contributes to a new dimension of knowledge on AAA pathogenesis. In this study, through matching conserved seed sequence, the circRNA/miRNA interaction networks were constructed according to miRNA binding sites. Among the circRNA/miRNA interaction networks, hsa_circ_0005360 and hsa_circ_0002168 harbors one binding site with miR-181b and miR-15a, respectively. Remarkably, miR-181b was determined highly expressed in human AAA and correlated with decreased expression of tissue inhibitor of metalloproteinase-3 and elastin, which promoted the progression of AAA [28]. In addition, miR-15a was reported a negative regulatory role in the expression of CDKN2B and thus promoting the apoptosis of VSMC, which might lead to the pathogenesis of AAA [35]. Further studies are warranted to confirm the hsa_circ_0005360/miR-181b and hsa_circ_0002168/miR-15a axis in AAA.

This study has several potential limitations. First, the sample size is relatively small and the results should be cautious to interpret. A multicenter study with large sample size may reduce the ethical biases and improve the reliability of the microarray data. Second, not all circRNAs function as the inhibitor of miRNAs. In fact, circRNAs can also interact with RNA-binding proteins, modulate transcription and alternative splicing, and even be translated, which are not mentioned in this study. Third, all the functional annotation of circRNAs and interaction networks were predicted based on bioinformatics analysis. Further experimental studies are warranted to clarify the potential mechanisms.

## Conclusions

In summary, the dysregulated circRNAs identified by our study may have a regulative role in the initiation and progression of AAA. Additionally, circRNA/miRNA interaction networks provide new insights into the molecular mechanisms and potential therapeutic targets for AAA.

## Data Availability

The datasets generated and/or analyzed during the current study are available in the Gene Expression Omnibus (GEO, http://www.ncbi.nlm.nih.gov/geo/) database (Accession Number: GSE144431).

## Abbreviations

AAA: Abdominal aortic aneurysm
BP: Biological process
CC: Cellular component
circRNAs: Circular RNAs
cRNA: Complementary RNA
GO: Gene ontology
hsa: *Homo sapiens*
KEGG: Kyoto encyclopedia of genes and genomes
MF: Molecular function
mRNA: Messenger RNA
qRT-PCR: Quantitative real-time polymerase chain reaction.

## References

[1] Wilmink AB, Quick CR (1998). Epidemiology and potential for prevention of abdominal aortic aneurysm. Br J Surg.

[2] Johansson M, Zahl PH, Siersma V, Jorgensen KJ, Marklund B, Brodersen J (2018). Benefits and harms of screening men for abdominal aortic aneurysm in Sweden: a registry-based cohort study. Lancet.

[3] Li J, Pan C, Zhang S, Spin JM, Deng A, Leung LLK, Dalman RL, Tsao PS, Snyder M (2018). Decoding the genomics of abdominal aortic aneurysm. Cell.

[4] Wilusz JE, Sharp PA (2013). Molecular biology. A circuitous route to noncoding RNA. Science.

[5] Conn SJ, Pillman KA, Toubia J, Conn VM, Salmanidis M, Phillips CA, Roslan S, Schreiber AW, Gregory PA, Goodall GJ (2015). The RNA binding protein quaking regulates formation of circRNAs. Cell.

[6] Memczak S, Jens M, Elefsinioti A, Torti F, Krueger J, Rybak A, Maier L, Mackowiak SD, Gregersen LH, Munschauer M (2013). Circular RNAs are a large class of animal RNAs with regulatory potency. Nature.

[7] Zhang Z, Xie Q, He D, Ling Y, Li Y, Li J, Zhang H (2018). Circular RNA: new star, new hope in cancer. BMC Cancer.

[8] Barrett SP, Salzman J (2016). Circular RNAs: analysis, expression and potential functions. Development.

[9] Holdt LM, Stahringer A, Sass K, Pichler G, Kulak NA, Wilfert W, Kohlmaier A, Herbst A, Northoff BH, Nicolaou A (2016). Circular non-coding RNA ANRIL modulates ribosomal RNA maturation and atherosclerosis in humans. Nat Commun.

[10] Vausort M, Salgado-Somoza A, Zhang L, Leszek P, Scholz M, Teren A, Burkhardt R, Thiery J, Wagner DR, Devaux Y (2016). Myocardial infarction-associated circular RNA predicting left ventricular dysfunction. J Am Coll Cardiol.

[11] Tan WL, Lim BT, Anene-Nzelu CG, Ackers-Johnson M, Dashi A, See K, Tiang Z, Lee DP, Chua WW, Luu TD (2017). A landscape of circular RNA expression in the human heart. Cardiovasc Res.

[12] Enright AJ, John B, Gaul U, Tuschl T, Sander C, Marks DS (2003). MicroRNA targets in drosophila. Genome Biol.

[13] Pasquinelli AE (2012). MicroRNAs and their targets: recognition, regulation and an emerging reciprocal relationship. Nat Rev Genet.

[14] Huang da W, Sherman BT, Lempicki RA (2009). Systematic and integrative analysis of large gene lists using DAVID bioinformatics resources. Nat Protoc.

[15] Schmittgen TD, Livak KJ (2008). Analyzing real-time PCR data by the comparative C(T) method. Nat Protoc.

[16] Jones GT, Tromp G, Kuivaniemi H, Gretarsdottir S, Baas AF, Giusti B, Strauss E, Van't Hof FN, Webb TR, Erdman R (2017). Meta-analysis of genome-wide association studies for abdominal aortic aneurysm identifies four new disease-specific risk loci. Circ Res.

[17] Sakalihasan N, Michel JB, Katsargyris A, Kuivaniemi H, Defraigne JO, Nchimi A, Powell JT, Yoshimura K, Hultgren R (2018). Abdominal aortic aneurysms. Nat Rev Dis Primers.

[18] Thompson RW (2002). Reflections on the pathogenesis of abdominal aortic aneurysms. Cardiovasc Surg.

[19] Brady AR, Thompson SG, Fowkes FG, Greenhalgh RM, Powell JT, Participants UKSAT (2004). Abdominal aortic aneurysm expansion: risk factors and time intervals for surveillance. Circulation.

[20] Michel JB, Martin-Ventura JL, Egido J, Sakalihasan N, Treska V, Lindholt J, Allaire E, Thorsteinsdottir U, Cockerill G, Swedenborg J (2011). Novel aspects of the pathogenesis of aneurysms of the abdominal aorta in humans. Cardiovasc Res.

[21] Palazzuoli A, Gallotta M, Guerrieri G, Quatrini I, Franci B, Campagna MS, Neri E, Benvenuti A, Sassi C, Nuti R (2008). Prevalence of risk factors, coronary and systemic atherosclerosis in abdominal aortic aneurysm: comparison with high cardiovascular risk population. Vasc Health Risk Manag.

[22] Biros E, Gabel G, Moran CS, Schreurs C, Lindeman JH, Walker PJ, Nataatmadja M, West M, Holdt LM, Hinterseher I (2015). Differential gene expression in human abdominal aortic aneurysm and aortic occlusive disease. Oncotarget.

[23] Lenk GM, Tromp G, Weinsheimer S, Gatalica Z, Berguer R, Kuivaniemi H (2007). Whole genome expression profiling reveals a significant role for immune function in human abdominal aortic aneurysms. BMC Genomics.

[24] Gabel G, Northoff BH, Weinzierl I, Ludwig S, Hinterseher I, Wilfert W, Teupser D, Doderer SA, Bergert H, Schonleben F, et al. Molecular Fingerprint for Terminal Abdominal Aortic Aneurysm Disease. J Am Heart Assoc. 2017;6(12). 10.1161/JAHA.117.006798. 10.1161/JAHA.117.006798 PMC5779007 29191809

[25] Yang YG, Li MX, Kou L, Zhou Y, Qin YW, Liu XJ, Chen Z (2016). Long noncoding RNA expression signatures of abdominal aortic aneurysm revealed by microarray. Biomed Environ Sci.

[26] Wang J, Sun H, Zhou Y, Huang K, Que J, Peng Y, Wang J, Lin C, Xue Y, Ji K (2019). Circular RNA microarray expression profile in 3,4-benzopyrene/angiotensin II-induced abdominal aortic aneurysm in mice. J Cell Biochem.

[27] Bradley DT, Hughes AE, Badger SA, Jones GT, Harrison SC, Wright BJ, Bumpstead S, Baas AF, Gretarsdottir S, Burnand K (2013). A variant in LDLR is associated with abdominal aortic aneurysm. Circ Cardiovasc Genet.

[28] Di Gregoli K, Mohamad Anuar NN, Bianco R, White SJ, Newby AC, George SJ, Johnson JL (2017). MicroRNA-181b controls atherosclerosis and aneurysms through regulation of TIMP-3 and elastin. Circ Res.

[29] Harris D, Liang Y, Chen C, Li S, Patel O, Qin Z (2015). Bone marrow from blotchy mice is dispensable to regulate blood copper and aortic pathologies but required for inflammatory mediator production in LDLR-deficient mice during chronic angiotensin II infusion. Ann Vasc Surg.

[30] Thatcher SE, Zhang X, Howatt DA, Yiannikouris F, Gurley SB, Ennis T, Curci JA, Daugherty A, Cassis LA (2014). Angiotensin-converting enzyme 2 decreases formation and severity of angiotensin II-induced abdominal aortic aneurysms. Arterioscler Thromb Vasc Biol.

[31] Zheng C, Niu H, Li M, Zhang H, Yang Z, Tian L, Wu Z, Li D, Chen X (2015). Cyclic RNA hsacirc000595 regulates apoptosis of aortic smooth muscle cells. Mol Med Rep.

[32] Chen J, Cui L, Yuan J, Zhang Y, Sang H (2017). Circular RNA WDR77 target FGF-2 to regulate vascular smooth muscle cells proliferation and migration by sponging miR-124. Biochem Biophys Res Commun.

[33] Mao YY, Wang JQ, Guo XX, Bi Y, Wang CX (2018). Circ-SATB2 upregulates STIM1 expression and regulates vascular smooth muscle cell proliferation and differentiation through miR-939. Biochem Biophys Res Commun.

[34] Dang RY, Liu FL, Li Y (2017). Circular RNA hsa_circ_0010729 regulates vascular endothelial cell proliferation and apoptosis by targeting the miR-186/HIF-1alpha axis. Biochem Biophys Res Commun.

[35] Gao P, Si J, Yang B, Yu J (2017). Upregulation of MicroRNA-15a contributes to pathogenesis of abdominal aortic aneurysm (AAA) by modulating the expression of Cyclin-dependent kinase inhibitor 2B (CDKN2B). Med Sci Monit.

